# Incidence and Antibiotic Susceptibility of Gram‐Negative Bacteria Associated With Chest Infections in Intensive Care Unit Patients From a Selected Hospital in the Kingdom of Saudi Arabia

**DOI:** 10.1155/bmri/5283526

**Published:** 2025-11-28

**Authors:** Afrah Almouwlid, Kamal Albenasy, Yasser Kamel, Abdelrahman Abdelmoktader, Mohammed Alaidarous, Ahmed Abdel-Hadi

**Affiliations:** ^1^ Department of Medical Laboratory Sciences, College of Applied Medical Sciences, Majmaah University, Majmaah, Saudi Arabia, mu.edu.sa; ^2^ Faculty of Medicine, King Abdulaziz University, Jeddah, Saudi Arabia, kau.edu.sa; ^3^ Microbiology Department, Faculty of Medicine, Fayoum University, Fayoum, Egypt, fayoum.edu.eg

**Keywords:** antimicrobial resistance, carbapenem resistance, Gram-negative bacilli, hospital-acquired pneumonia, ICU, multidrug-resistant organisms, Saudi Arabia, ventilator-associated pneumonia

## Abstract

**Background:**

Hospital‐acquired pneumonia (HAP), including ventilator‐associated pneumonia (VAP), is a leading cause of morbidity and mortality in intensive care units (ICUs). Local, organism‐specific antimicrobial resistance data are critical to guide empiric therapy and strengthen antimicrobial stewardship efforts.

**Objective:**

The aim of this study is to describe the spectrum of Gram‐negative bacilli (GNB) responsible for ICU‐acquired lower respiratory tract infections (LRTIs) in a Saudi general hospital and to characterize their antimicrobial resistance profiles, including multidrug‐resistant (MDR), extensively drug‐resistant (XDR), and pandrug‐resistant (PDR) patterns.

**Methods:**

We retrospectively analyzed 271 nonduplicate GNB isolates recovered from ICU respiratory specimens (sputum, tracheal aspirates, and throat swabs) collected between 2020 and 2022. Demographic characteristics, specimen distribution, bacterial species, and antimicrobial susceptibility patterns were summarized. MDR, XDR, and PDR classifications were determined according to standard phenotypic criteria.

**Results:**

Of the 271 specimens, 126 (46%) were sputum, 108 (40%) were tracheal aspirates, and 37 (14%) were throat swabs. Patients were 52% male (141/271) and 48% female (130/271), with 56% aged > 65 years. Twenty‐three GNB species were identified; the predominant pathogens were *Klebsiella* spp. (92/271, 34.0%), *Pseudomonas* spp. (73/271, 27.0%), and *Acinetobacter* spp. (32/271, 12%). Enterobacteriaceae accounted for 130 isolates (48.0%), while non‐Enterobacteriaceae comprised 141 (52.0%). There were statistically significant (*p* = 0.016) differences between the three most common organisms (*Klebsiella pneumoniae*, *Pseudomonas aeruginosa*, and *Acinetobacter baumannii* complex). Antimicrobial susceptibility testing revealed extensive resistance patterns across the major isolates. *Pseudomonas* spp. demonstrated very high resistance to cephalosporins (> 95%), with lower resistance observed to amikacin (43%). *Acinetobacter* spp. showed the most alarming profile, with nearly universal resistance to *β*‐lactams and carbapenems (> 90%), although colistin retained complete activity (0% resistance). In contrast, *Klebsiella* spp. exhibited high resistance to third‐generation cephalosporins (86%–93%) and carbapenems (70%–77%) while maintaining moderate susceptibility to amikacin (45%) and tigecycline (36%). These findings demonstrate a substantial burden of MDR among ICU GNB isolates, with colistin emerging as the only consistently effective therapeutic option.

**Conclusions:**

ICU cohort is dominated by a limited number of highly resistant GNB led by *K. pneumoniae*, *P. aeruginosa*, and *A. baumannii*. The cohort predominantly affects older adults (> 60 years), and the breadth of MDR/XDR/PDR underscores the urgency of rigorous antimicrobial stewardship, infection prevention, and rapid diagnostics to optimize empiric therapy.

## 1. Introduction

Hospital‐acquired pneumonia (HAP), including ventilator‐associated pneumonia (VAP), is one of the most consequential infections in intensive care units (ICUs) [1]. The clinical challenge is twofold: first, timely selection of empiric therapy for critically ill patients who can deteriorate rapidly; second, escalating antimicrobial resistance that progressively erodes the effectiveness of traditional regimens [2].

Lower respiratory tract infections (LRTIs) remain a leading cause of morbidity and mortality in critically ill patients worldwide, particularly in ICUs, where VAP and HAP constitute a major burden [3].

These infections are often caused by Gram‐negative bacilli (GNB), which are recognized as the most significant contributors to ICU‐acquired pneumonia due to their intrinsic resistance mechanisms, ability to survive in hospital environments, and high potential for nosocomial transmission [4]. Among these, *Klebsiella pneumoniae*, *Pseudomonas aeruginosa*, and *Acinetobacter baumannii* are consistently identified as the predominant pathogens [5].

The emergence and spread of antimicrobial resistance among GNB have become a pressing global health concern. Of particular importance is the rise of multidrug resistance (MDR), extensively drug‐resistant (XDR), and pandrug resistance (PDR), which severely limit therapeutic options and are associated with poor clinical outcomes [6]. Multidrug‐resistant organisms (MDROs) are defined by resistance to at least three antimicrobial classes, while XDR isolates remain susceptible to only one or two categories, and PDR strains are resistant to all available classes. These definitions highlight the continuum of resistance that complicates clinical management in ICU settings [7].

Globally, the increasing incidence of carbapenem‐resistant Enterobacteriaceae, carbapenem‐resistant *A. baumannii*, and MDR *P. aeruginosa* has been recognized by the World Health Organization [8] as a critical priority for research and drug development. In the Middle East, including Saudi Arabia, several studies have documented alarmingly high rates of resistance among ICU pathogens, reflecting both local antimicrobial prescribing practices and broader global trends [9]. The widespread misuse of broad‐spectrum antibiotics, combined with prolonged ICU stays, mechanical ventilation, and invasive procedures, contributes to the selection and dissemination of resistant strains [10].

In Saudi Arabia, data on the prevalence and resistance profiles of GNB in ICU‐acquired pneumonia remain limited [9], particularly in peripheral regions outside major urban centers. Understanding the local epidemiology of pathogens and their resistance patterns is essential for guiding empiric therapy, optimizing antimicrobial stewardship, and informing infection prevention strategies.

The present study, therefore, is aimed at describing the prevalence, species distribution, and antimicrobial resistance profiles of GNB isolated from ICU‐acquired LRTIs over 3 years in Rabigh General Hospital, Saudi Arabia. Particular emphasis is placed on the burden of MDR, XDR, and PDR strains to contribute to local surveillance data and inform evidence‐based clinical decision‐making.

## 2. Materials and Methods

This investigation was a retrospective cross‐sectional study conducted in the ICU of Rabigh General Hospital, located in the western region of the Kingdom of Saudi Arabia. The hospital is a government tertiary‐care facility that provides comprehensive health services to the city of Rabigh and nearby communities. The ICU, comprising eight beds across two sections, admits critically ill patients with a wide range of medical and surgical conditions. Approximately 70 patients are served each month.

The study period covered January 2020 through December 2022, during which respiratory specimens were collected from patients with clinically suspected LRTI, including HAP and VAP.

### 2.1. Study Population

All ICU patients admitted during the study period and diagnosed with pneumonia based on clinical, radiological, and microbiological criteria were considered for inclusion. Both male and female patients of all ages were eligible.

#### 2.1.1. Inclusion Criteria

• Patients admitted to the ICU during the study period.
• Clinical diagnosis of pneumonia (new or progressive infiltrates on chest imaging plus compatible signs/symptoms such as fever, purulent sputum, leukocytosis/leukopenia, or hypoxia).
• Positive culture for GNB from respiratory specimens (sputum, tracheal aspirate, or throat swab).

#### 2.1.2. Exclusion Criteria

• Patients with postoperative surgical complications without evidence of pneumonia.
• Cultures yielding Gram‐positive organisms, fungi, or contaminants without GNB.
• Duplicate isolates from the same patient episode.

### 2.2. Sample Collection

A total of 271 nonduplicate GNB isolates were included. Specimen types comprised sputum (126 samples, 46%); tracheal aspirates (108 samples, 40%), and throat swabs (37 samples, 14%). Specimens were collected under aseptic conditions by trained ICU staff and immediately transported to the microbiology laboratory for processing.

### 2.3. Microbiological Processing

#### 2.3.1. Identification of Isolates

Bacterial isolates were cultured using P50 plates for GNB and identified in the laboratory using the MicroScan WalkAway Plus System (Beckman Coulter, Version 3960047) [11], which performs biochemical profiling for species‐level identification.

#### 2.3.2. Antimicrobial Susceptibility Testing (AST)

AST was performed using the same MicroScan platform according to the manufacturer′s protocols. Results were interpreted following Clinical and Laboratory Standards Institute (CLSI) (2020) guidelines [11]. The antibiotic classes tested included aminoglycosides (amikacin, gentamicin, and tobramycin), *β*‐lactam/*β*‐lactamase inhibitors (amoxicillin–clavulanic acid, ampicillin–sulbactam, and piperacillin–tazobactam), penicillins and monobactams (ampicillin and aztreonam), cephalosporins (cefazolin, cefoxitin, cefuroxime, cefotaxime, ceftazidime, and cefepime [with and without clavulanate combinations]), carbapenems (ertapenem, imipenem, and meropenem), fluoroquinolones (ciprofloxacin, levofloxacin, norfloxacin, and moxifloxacin), and other classes (nitrofurantoin, colistin, tigecycline, and trimethoprim–sulfamethoxazole).

#### 2.3.3. Definitions of Resistance Categories

Phenotypic classifications were applied according to standard definitions [7]: MDR: nonsusceptibility to at least one agent in three or more antimicrobial categories. XDR: nonsusceptibility to at least one agent in all but two or fewer categories. PDR: nonsusceptibility to all agents in all tested categories. Resistance mechanisms of interest included extended‐spectrum beta‐lactamases (ESBLs), metallo‐beta‐lactamases (MBLs), and carbapenemase‐producing organisms (CPOs), inferred from phenotypic resistance profiles.

### 2.4. Statistical Analysis

Patient demographic data, specimen types, microbial identification, and AST results were entered into a database. Analysis was performed using IBM SPSS Statistics, Version 27 (IBM Corp., Armonk, NY, United States) [12]. Descriptive statistics (frequency and percentage) were used for categorical variables. Chi‐square test was applied to evaluate associations between categorical variables (e.g., resistance rates by species, sex, or age group). Fisher′s exact test was used for 2 × 2 comparisons with small expected counts. A *p* value < 0.05 was considered statistically significant.

### 2.5. Ethical Considerations

The study was approved by the National Committee of Bioethics (NCBE), Jeddah (Approval No. A01581) [13]. Administrative approval was obtained from the Directorate of Health Affairs, Jeddah, and the hospital′s medical administration. Informed consent was waived due to the retrospective design; however, all patient data were anonymized and handled with strict confidentiality.

## 3. Results

### 3.1. Demographic Characteristics

A total of 271 patients admitted to the ICU between January 2020 and December 2022 were included. Of these, 141 (52%) were male and 130 (48%) were female. The majority of cases occurred among patients aged > 65 years (152/271, 56%) compared to ≤ 65 years (119/271, 44%) (Table 1).

**Table 1:** Age and gender distribution of collected samples from patients in intensive care units (*n* = 271).

**Sex**	**≤ 65 years**	**> 65 years**	**Total**
Male	58	83	141(%52)
Female	61	69	130 (%48)
Total	119 (44%)	152 (56%)	271 (100%)

### 3.2. Clinical Specimens

Among the 271 respiratory samples, sputum (126, 46%) was the most common specimen type, followed by tracheal aspirates (108, 40%) and throat swabs (37, 14%). Enterobacteriaceae were more frequent in throat swabs, while nonfermenters were prominent in tracheal aspirates and sputum (Table 2).

**Table 2:** Frequency of Enterobacteriaceae pathogens isolated from different clinical samples of patients in intensive care units.

**Clinical samples**	**Total no. of samples**	**Total no. of infected samples**	**The number (%) of bacterial isolates found in each clinical samples**								
			***K. pneumoniae***	***K. oxytoca***	***K. ozaenae***	***E. cloacae***	***E. aerogenes***	***Proteus mirabilis***	***E. coli***	***Citrobacter freundii***	***Providencia rustigianii***
Sputum	126	52	30	0	0	6	2	6	4	3	1
	46.5%	40.3%	23.8%	0%	0%	4.8%	1.6%	4.8%	3.2%	2.4%	0.8%
Tracheal aspirate	108	56	43	1	0	1	1	7	3	0	0
	40%	51.9%	39.8%	0.9%	0%	0.9%	0.9%	6.5%	2.7%	0%	0%
Throat	37	22	17	0	1	4	0	0	0	0	0
	13.5%	59.5%	45.9%	0%	2.7%	10.8%	0%	0%	0%	0%	0%
Total	271	130	90	1	1	11	3	13	7	3	1
		48%	33.2%	0.4%	0.4%	4%	1.2%	4.8%	2.6%	1.2%	0.4%

### 3.3. Bacterial Species Distribution

The samples were split into two groups based on the type of isolated bacteria: the first group included 130 (48%) Enterobacteriaceae, comprising 52 sputum samples, 56 tracheal aspirates, and 22 throat samples (Table 2). The second group included 141 non‐Enterobacteriaceae bacteria, comprising 73 sputum samples, 53 tracheal aspirates, and 15 throat samples (Table 3). The predominant species were from the *Enterobacteriaceae* family, with *K. pneumoniae* being the most frequently isolated (90, 33.2%), followed by *Proteus mirabilis* (13, 4.8%), *Enterobacter cloacae* (11, 4%), and *Escherichia coli* (7, 2.6%). Additionally, from the non‐*Enterobacteriaceae* group, *P. aeruginosa* (69, 25.5%) and *A. baumannii* complex (31, 11.4%) were isolated. The frequency of *K. pneumoniae* was highest in throat samples (45.9%), followed by tracheal aspirates (39.8%) and sputum (23.8%), while *P. aeruginosa* was highest in sputum (32.5%), followed by tracheal aspirates (23.1%) and throat samples (8%). Additionally, the *A. baumannii* complex was highest in throat samples (16.2%), followed by tracheal aspirates (12%) and sputum (9.5%).

**Table 3:** Frequency of non‐Enterobacteriaceae pathogens isolated from different clinical samples of patients in intensive care units.

**Clinical samples**	**Total no. of samples**	**Total no. of infected samples**	**The number (%) of bacterial isolates found in each clinical samples**													
			***P. aeruginosa***	***P. fluorescens***	***A. baumannii* complex**	***A. lwoffii***	***Burkholderia cepacia* complex**	***Stenotrophomonas maltophilia***	***Chryseobacterium indologenes***	***A. xylosoxidans/denitrificans***	***A. piechaudii***	***Providencia stuartii***	***Bordetella bronchiseptica***	***Morganella morganii***	***Serratia marcescens***	***Wautersiella falsenii***
Sputum	126	73	41	3	12	0	10	2	1	1	1	1	0	1	0	0
	46.5%	57.9%	32.5%	2.4%	9.5%	0%	8%	1.6%	0.8%	0.8%	0.8%	0.8%	0%	0.8%	0%	0%
Tracheal aspirate	108	53	25	0	13	0	5	4	3	0	0	1	0	0	1	1
	40%	49.1%	23.1%	0%	12%	0%	4.6%	3.7%	2.8%	0%	0%	0.9%	0%	0%	0.9%	0.9%
Throat	37	15	3	1	6	1	0	2	0	1	0	0	1	0	0	0
	13.5%	40.5%	8.1%	2.7%	16.2%	2.7%	0%	5.4%	0%	2.7%	0%	0%	2.7%	0%	0%	0%
Total	271	141	69	4	31	1	15	8	4	2	1	2	1	1	1	1
		52%	25.5%	1.5%	11.4%	0.4%	5.5%	3%	1.5%	0.8%	0.4%	0.8%	0.4%	0.4%	0.4%	0.4%

### 3.4. Antimicrobial Susceptibility Patterns

Among Enterobacteriaceae, *Klebsiella* species exhibited the highest resistance to colistin (87%), followed by third‐generation cephalosporins: cefotaxime (76%), ceftazidime (71%), and cefepime (68%). Resistance to fluoroquinolones was also notable: ciprofloxacin (68%) and levofloxacin (61%). Moderate resistance was observed for aminoglycosides: amikacin (53%) and gentamicin (60%), as well as for carbapenems: ertapenem (64%), imipenem (57%), and meropenem (57%). Tigecycline demonstrated the lowest resistance (36%) (Table 4).

**Table 4:** Antimicrobial susceptibility profile of *Klebsiella* spp. (*n* = 92) among Enterobacteriaceae.

**N**	**Antibiotic**		***Klebsiella* species (** **n** = 92**)**		
			**Sensitive (%)**	**Intermediate (%)**	**Resistance (%)**
	**Group**	**Item**			
1	Aminoglycosides	Amikacin	39 (42%)	4 (4%)	49 (53%)
2		Gentamicin	37 (40%)	0 (0%)	55 (60%)

3	*β*‐Lactams	Cefotaxime	21 (23%)	1 (1%)	70 (76%)
4		Ceftazidime	27 (29%)	0 (0%)	65 (71%)
5		Cefepime	27 (29%)	3 (3%)	62 (68%)

6	Carbapenems	Ertapenem	31 (33%)	2 (3%)	59 (64%)
7		Imipenem	33 (36%)	7 (7%)	52 (57%)
8		Meropenem	39 (42%)	1 (1%)	52 (57%)

9	Fluoroquinolones	Ciprofloxacin	28 (30%)	2 (2%)	62 (68%)
10		Levofloxacin	32 (35%)	4 (4%)	56 (61%)

11	Polymyxins	Colistin	0 (0%)	12 (13%)	80 (87%)
12	Tigecycline	Tigecycline	41 (45%)	18 (19%)	33 (36%)

Nonfermenters such as *P. aeruginosa* and *A. baumannii* exhibited widespread resistance to *β*‐lactams, with aztreonam showing the highest resistance (92%), followed by cefepime (89%), ceftazidime (86%), and piperacillin/tazobactam (86%). Resistance to carbapenems ranged from 62% (meropenem) to 78% (imipenem). Fluoroquinolone resistance was lower: ciprofloxacin (44%) and levofloxacin (41%). Aminoglycosides showed moderate resistance (amikacin 42%, gentamicin 48%, and tobramycin 45%), while colistin remained active against most isolates (63% resistance) (Tables 5 and 6).

**Table 5:** Antimicrobial susceptibility profile of *Acinetobacter* spp. (*n* = 32) among non‐Enterobacteriaceae.

	**Antibiotic**		***Acinetobacter* spp. (** **n** = 32**)**		
	**Group**	**Item**	**Sensitive,** **n** **(%)**	**Intermediate,** **n** **(%)**	**Resistance,** **n** **(%)**
1	Aminoglycosides	Amikacin	11 (34%)	2 (7%)	19 (59%)
2		Gentamicin	15 (47%)	3 (9%)	14 (44%)

3	*β*‐Lactams	Ampicillin–sulbactam	0 (0%)	2 (6%)	30 (94%)
4		Ceftazidime	8 (25%)	1 (3%)	23 (72%)
5		Cefepime	10 (31%)	1 (3%)	21 (66%)

6	Carbapenems	Imipenem	0 (0%)	0 (0%)	32 (100%)
7		Meropenem	10 (31%)	0 (0%)	22 (69%)

8	Fluoroquinolones	Ciprofloxacin	10 (31%)	0 (0%)	22 (69%)
9		Levofloxacin	10 (31%)	2 (6%)	20 (63%)
10	Tigecycline	Tigecycline	0 (0%)	1 (3%)	31 (97%)

**Table 6:** Antimicrobial susceptibility profile of *Pseudomonas* spp. (*n* = 73) among non‐Enterobacteriaceae.

	**Antibiotic**		***Pseudomonas* spp. (** **n** = 73**)**		
	**Group**	**Item**	**Sensitive,** **n** **(%)**	**Intermediate,** **n** **(%)**	**Resistance,** **n** **(%)**
1	Aminoglycosides	Amikacin	38 (52)	4 (6%)	31 (42%)
2		Gentamicin	22 (30%)	16 (22%)	35 (48%)
3		Tobramycin	36 (49%)	4 (6%)	33 (45%)

4	Antipseudomonal *β*‐lactams	Ceftazidime	6 (8%)	4 (6%)	63 (86%)
5		Cefepime	5 (7%)	3 (4%)	65 (89%)

6	Penicillin	Pip/tazo	5 (7%)	5 (7%)	63 (86%)
7	Monobactam	Aztreonam	5 (7%)	1 (1%)	67 (92%)

8	Carbapenems	Imipenem	10 (14%)	6 (8%)	57 (78%)
9		Meropenem	22 (30%)	6 (8%)	45 (62%)

10	Fluoroquinolones	Ciprofloxacin	37 (50%)	4 (6%)	32 (44%)
11		Levofloxacin	34 (47%)	9 (12%)	30 (41%)
12	Polymyxins	Colistin	13 (18%)	14 (19%)	46 (63%)

### 3.5. MDR, XDR, and PDR Distribution

As shown in Table 7, MDR was documented in 44% of all resistant isolates (*n* = 259). The highest proportion of MDR was observed in *Klebsiella* spp. (22%), followed by *Pseudomonas* spp. (15%) and *Acinetobacter* spp. (6%). XDR strains were detected in all three major pathogens, with 3% in *Klebsiella* spp., 8% in *Pseudomonas* spp., and 2% in *Acinetobacter* spp. PDR phenotypes were rare, accounting for 7% in *Klebsiella* spp., 5% in *Pseudomonas* spp., and 4% in *Acinetobacter* spp.

**Table 7:** Distribution of MDR, XDR, and PDR among major Gram‐negative bacterial isolates (*n* = 259).

**Pathogen**	**Resistance mechanisms (** **n** = 259**)**			**Total isolates (** **n** **, %)**
	**MDR (%)**	**XDR (%)**	**PDR (%)**	
*Klebsiella* spp.	57 (22%)	8 (3%)	17 (7%)	82 (32%)
*Pseudomonas* spp.	40 (15%)	21 (8%)	12 (5%)	73 (28%)
*Acinetobacter* spp.	16 (6%)	5 (2%)	11 (4%)	32 (12%)
Total	113 (44%)	34 (13%)	40 (15%)	187 (72%)

In Table 8, no significant sex differences were observed among the three organisms. However, age distribution differed significantly, with a higher proportion of children infected with *P. aeruginosa* compared to the *A. baumannii* complex and *K. pneumoniae*. Conversely, infections with *K. pneumoniae* were more common in older adults than in the other two organisms.

**Table 8:** Proportion of high‐rate Gram‐negative bacteria collected from ICU patients of all age and gender groups.

**Variable**	**Category**	**Types of bacteria**			**p value**
		***A. baumannii*, n(%)**	***K. pneumoniae*, n(%)**	***P. aeruginosa*, n(%)**	
Sex	Female	12 (38.7%)	49 (54.4%)	36 (52.2%)	0.311
	Male	19 (61.3%)	41 (45.6%)	33 (47.8%)	

Age group	< 18	4 (12.9%)	7 (7.8%)	19 (27.5%)	0.016
	18–60	9 (29.0%)	24 (26.7%)	17 (24.6%)	
	> 60	18 (58.1%)	59 (65.6%)	33 (47.8%)	

## 4. Discussion

The present study provides valuable insight into the epidemiology of GNB causing ICU‐acquired LRTIs in a Saudi tertiary hospital. The findings confirm that *K. pneumoniae*, *P. aeruginosa*, and *A. baumannii* remain the dominant pathogens in critically ill patients, together accounting for almost 70% of isolates.

This distribution mirrors observations from other hospitals in Saudi Arabia, Egypt, and Sudan, where *K. pneumoniae* consistently emerges as the most prevalent respiratory isolate, followed by *Pseudomonas* and *Acinetobacter* species. International reports from Europe and Asia also highlight these three organisms as critical‐priority pathogens, emphasizing their global significance in ICU‐acquired pneumonia [14–18]. A recent study from South Qunfudah Hospital, Saudi Arabia, confirmed the predominance of *K. pneumoniae* and noted an emerging antimicrobial resistance profile [19].

The majority of affected patients in our study were elderly, with more than half over the age of 65. This age group is particularly vulnerable due to multiple comorbidities, immunosenescence, and prolonged exposure to invasive devices and broad‐spectrum antibiotics. Older patients (> 65 years) were more frequently infected with resistant strains, particularly *K. pneumoniae* and *A. baumannii*. Chi‐square analysis confirmed significant associations between age group and bacterial species distribution (*p* < 0.016). No significant association was found between sex and resistance patterns.

Similar associations between advanced age and ICU pneumonia have been documented in studies [20, 21], which consistently demonstrate that elderly patients not only acquire infections more frequently but are also more likely to harbor resistant strains.

The relationship between *P. aeruginosa* infection and the respiratory system is not yet fully understood; however, several contributing factors have been identified. Children with neurological disorders, such as neuromuscular disorders (NMD) and cerebral palsy (CP), are more susceptible to severe respiratory infections [22, 23].

Previous research reported that among children with CP in the ICU, 89% were infected with *P. aeruginosa* or *Klebsiella* species, and the mortality rate was 39%, primarily due to pneumonia [24]. A more recent study conducted in 2023 indicated that 5.1% of children with CP had *P. aeruginosa* infection [25].

Furthermore, critically ill children with neurological disorders are particularly vulnerable to carbapenem‐resistant *P. aeruginosa* (CRPA). Evidence from a tertiary pediatric hospital in China showed that CRPA accounted for 18.4% of ICU infections, with prolonged hospital stay, invasive procedures, and recent blood transfusions identified as major risk factors [26]. These findings emphasize the need for early identification, strict infection control, and targeted antimicrobial strategies in this high‐risk population.

Both studies indicate that male ICU patients are more susceptible to VAP than females. A study published in 2023 in the Journal of Clinical Medicine reported that 63.3% of VAP cases occurred in males compared to 36.7% in females [27]. Similarly, a 2024 study in the Annals of Pulmonary and Critical Care Medicine found that the incidence among males was 64.1% versus 35.9% among females [28]. These findings confirm that gender represents an important risk factor for VAP in ICU settings, suggesting that biological and immunological differences between males and females may influence susceptibility [29].

Nonfermenters, particularly *A. baumannii*, exhibited very high resistance to *β*‐lactams, with carbapenem resistance ranging from 69% to 100%. Tigecycline showed the highest resistance rate (97%). These findings are consistent with previous surveillance programs in Saudi Arabia and regional studies from Oman and the United Arab Emirates, which describe *Acinetobacter* as a dominant carbapenem‐resistant organism [30, 31]. Recent local surveillance in Saudi Arabia further confirmed widespread carbapenem and MDR among ICU isolates of *A. baumannii* [32], while global reports emphasize its rising prevalence and clinical threat worldwide [33].

In Jeddah (2024), a clinical trial demonstrated that the novel antibiotic cefiderocol represents a promising therapeutic option against MDR isolates, particularly carbapenem‐resistant *K. pneumoniae* (CRKP) and CRPA, achieving high clinical cure rates [34]. These findings highlight the importance of cautiously adopting novel antimicrobials, with their use strictly guided by laboratory results as a last‐resort therapy [34]. Furthermore, another study emphasized the role of molecular diagnostic techniques, which have proven effective in Saudi Arabia by detecting key carbapenemase genes such as OXA‐48, NDM, and VIM in ICU patient isolates, thereby supporting early diagnosis and precise therapeutic decisions [35].

Additionally, a recent multicenter observational study in Morocco (2025) using multiplex PCR (mPCR) showed very high sensitivity and specificity (~96.9% and 92%, respectively) compared to conventional methods; treatment regimens were modified in about 58% of patients, the proportion of appropriate empiric therapy increased from ~38.7% to ~67%, and appropriate post‐mPCR therapy was associated with reduced ICU mortality [36].

*P. aeruginosa* showed slightly better susceptibility than *Acinetobacter* but still exhibited high resistance across multiple *β*‐lactams (86%–92%) and carbapenems (62%–78%). Aminoglycosides and fluoroquinolones displayed moderate resistance (41%–48%), while colistin was the only drug that retained relatively better activity (63%). This pattern reflects both regional and global observations, confirming that *Pseudomonas* retains a wider but progressively narrowing therapeutic window [37].

The overall prevalence of MDR among Gram‐negative isolates in our study was 44%, with *K. pneumoniae* accounting for the highest proportion (22%), followed by *P. aeruginosa* (15%) and *A. baumannii* (6%). XDR strains were detected in 13% of isolates and PDR strains in 15%. Comparable MDR burdens have been reported in ICUs in Egypt, Saudi Arabia, and Sudan, where more than 40% of Gram‐negative isolates are classified as MDR [38–40]. The presence of XDR and PDR strains in our cohort underscores the gravity of the situation, as these categories significantly restrict therapeutic options and are associated with higher mortality.

Efforts to tackle MDR *A. baumannii* vary between the United Kingdom and Egypt. The United Kingdom relies on advanced genomic tools and enhanced surveillance to track resistance and prevent outbreaks, whereas Egypt focuses on exploring natural products as alternative or adjunct therapies to overcome high drug resistance. These approaches highlight the need for both robust infection control and novel therapeutic strategies in regions with high MDR prevalence [17].

The clinical and public health implications of these findings are substantial. From a clinical standpoint, the high resistance rates drastically limit empiric treatment options, forcing clinicians to rely on last‐line agents such as colistin and tigecycline, both of which are associated with toxicity and suboptimal outcomes. From a public health perspective, these results highlight the urgent need for robust antimicrobial stewardship programs in ICU settings, routine local resistance surveillance to inform empiric guidelines, and strict infection prevention and control practices, including ventilator bundles, hand hygiene, and environmental cleaning.

## 5. Limitations

This study has limitations, including its retrospective design, single‐center setting, and focus on respiratory specimens only, without molecular confirmation of resistance genes or assessment of clinical outcomes. Nonetheless, the relatively large number of isolates and comprehensive AST enhances the reliability of the findings.

## 6. Conclusion

This study demonstrates that GNB, particularly *K. pneumoniae*, *P. aeruginosa*, and *A. baumannii*, are the leading causes of ICU‐acquired LRTIs in Rabigh General Hospital, Saudi Arabia. The majority of patients affected were elderly (> 65 years), highlighting their vulnerability to severe infection and colonization by resistant pathogens.

**Figure 1:**
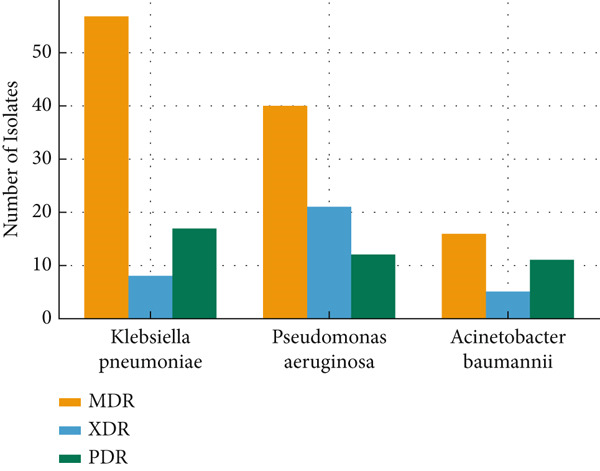
Distribution of MDR, XDR, and PDR among major Gram‐negative bacterial isolates (*n* = 259).

Antimicrobial resistance was widespread, with extremely high resistance to cephalosporins, fluoroquinolones, and nitrofurantoin and substantial resistance even to carbapenems. A worrying proportion of isolates exhibited MDR, while XDR and PDR phenotypes, although less common, were also detected (Figure 1). These findings align with both regional and global reports, underscoring that antimicrobial resistance among ICU Gram‐negative pathogens is a universal and escalating crisis.

The clinical implications are profound: empiric therapy in ICUs must be guided by local resistance surveillance and paired with rigorous antimicrobial stewardship programs to limit inappropriate antibiotic use. At the same time, infection prevention measures—including ventilator care bundles, strict hand hygiene, and environmental decontamination—are critical to preventing cross‐transmission within ICU environments.

In conclusion, the data from this study provide essential baseline information for clinicians and policymakers in Saudi Arabia. They highlight the urgent need for ongoing surveillance, strengthened stewardship, rapid diagnostics, and investment in new therapeutic options to combat the rising tide of drug‐resistant Gram‐negative infections in critical care settings.

## Disclosure

All authors have read and agreed to the published version of the manuscript.

## Conflicts of Interest

The authors declare no conflicts of interest.

## Author Contributions

Afrah Almouwlid: methodology, investigation, and analysis. Kamal Albenasy: conceptualization. Yasser Kamel: conceptualization, methodology, and data analysis. Abdelrahman Abdelmoktader: data analysis and validation. Mohammed Alaidarous: writing—review and editing. Ahmed Abdel‐Hadi: writing—review and editing and validation.

## Funding

This study was funded by the Deanship of Postgraduate Studies and Scientific Research at Majmaah University under Project Number R‐2025‐2090.

## Data Availability

The data that support the findings of this study are available on request from the corresponding author. The data are not publicly available due to privacy or ethical restrictions.
